# Incremental Hemodialysis: Review of Clinical Trials Focused on Patients Undergoing Once-Weekly Hemodialysis

**DOI:** 10.3390/nu17040713

**Published:** 2025-02-17

**Authors:** Piergiorgio Bolasco

**Affiliations:** Working Group for the Conservative Treatment of Chronic Kidney Failure of the Italian Society of Nephrology, 00185 Rome, Italy; pg.bolasco@gmail.com

**Keywords:** nutrition in hemodialysis, incremental hemodialysis, once-weekly hemodialysis

## Abstract

**Background/objectives**: The implementation of appropriate hemodialysis treatment in the transition from end-stage kidney disease to reduced frequency schedules represents a major challenge. The aim of our work is to report only treatment protocols that used once-weekly hemodialysis. **Methods**: The benefits and risks of 1WHD were explored in this systematic review. A search of MEDLINE, Scopus, and the Cochrane Central Register was conducted to identify publications relating to once-weekly hemodialysis trials performed between June 1981 and December 2024 and assess clinical impact, duration, safety, and mortality. Items, including age, causes of chronic kidney disease (CKD), creatinine levels, Blood Urea Nitrogen and GFR values, diuresis, nutritional supplementation, drop-out, survival, clinical benefit or drawbacks, and data from eventual control groups relating to higher frequency weekly HD sessions were included. Outcome at the end of a 1WHD regimen was represented by death or transition to twice/thrice-weekly HD rhythms. **Results**: A total of 1238 articles focused on IHD were included in the review, and 1226 trials were excluded as they referred either to twice-weekly hemodialysis (2WHD) schedules or failed to meet eligibility criteria, whilst another two were excluded based on incomplete outcome or patient recruitment issues. A total of eight articles comprising 254 patients undergoing 1WHD schedules were ultimately identified and evaluated. Only three studies focused on a comparison with a 1WHD schedule, whilst 107 referred to thrice-weekly HD (3WHD) and 15 2WHD). This choice demonstrated the possibility of slowing down the progression of CKD in the patients studied. Daily amino acid supplementation also proved to be beneficial. However, the milestone on which the 1WHD protocol is based is a low-protein diet. **Conclusions**: 1WHD has been shown to be safe and may result in improved clinical outcomes, particularly in appropriately selected patients. Large-scale randomized controlled trials should be carried out to confirm these potential advantages. However, the standard recruitment techniques applied tended to prevent suitably selected patients from transitioning into less frequent and potentially long-lasting 1WHD schedules.

## 1. Introduction

The concept of incremental dialysis was initially established in the context of peritoneal dialysis treatment [1]. Subsequently, the use of this term was extended to extra-corporeal dialysis, becoming known as incremental hemodialysis (IHD). IHD was adopted by a wide population of patients with End Stage Chronic Kidney Disease (ESKD) who were mainly treated through twice-weekly hemodialysis sessions (2WHD) until the need arose to progress to a thrice-weekly frequency (3WHD) [2,3,4,5,6,7]. One of the major benefits to patients on IHD is the choice of once-weekly hemodialysis (1WHD) characterized by a soft and better tolerated HD; the limits are rigorous selection of patients with an RKF between 6 and 10 mL/min/1.73 m^2^ and with a good adherence to a low-protein diet of between 0.4 and 0.6 g/kg. Conversely, in the 1WHD protocol with a period of six non-hemodialysis days, conservative and tailored nutrition plays a fundamental role in preserving residual renal function and modulating the inflammatory response in patients undergoing 1WHD until patients transition to a higher frequency of treatments. The practical experiences first described by Mitch et al. [8] and Morelli et al. [9] in the 1980s were reintroduced in the 1990s by Locatelli et al. with his Integrated Diet Dialysis Protocol (IDDP) [10,11]. The IDDP was known as the ‘Combined Diet Dialysis Program’ (CDDP) [12,13,14,15]. However, today, the majority of studies tend to prescribe a twice-weekly IHD schedule without an integrated low-protein nutritional program, relying merely on the persistence of adequate residual kidney function (RKF) and optimal efficiency of extracorporeal clearance aimed at slowing down the inevitable loss of glomerular filtration (GFR) and diuresis [16,17,18,19]. Indeed, even following the advent of 1WHD, many studies failed to integrate the schedule with a low-protein diet, or, even when doing so, tended to adopt a Very Low Protein Diet (VLPD) accounting for 0.3–0.4 g protein/kg/day with supplementation of amino acids and/or their keto-analogues, in the same way as for IDDP [10,11]. Integrated protocols based on the use of a VLPD put patients at higher risk of malnutrition, poor control of uremia, and low patient adherence due, in particular, to the large number of tablets or sachets to be taken daily. However, several studies opted for diets based on a higher protein content of 0.6 g protein/Kg/day (Low Protein Diet, LPD) together with supplementation of amino acids and/or their keto-analogues [12,13,14,15,16,17,18,19,20]. Lower-frequency hemodialysis schedule associated with a prudent but rigorously adhered to, low-protein diet is linked to a series of fundamental factors that affect mortality, morbidity, and quality of life of patients, i.e., (a) the well-known slower rate of RKF decline elicited by a low-protein diet, especially on 1WHD [21,22,23]; (b) a lower number of hemodialysis sessions with the consequent decrease in detrimental dialysis-induced hypercatabolic and inflammatory response resulting in clinical frailty of patients [24]—this situation can be improved by using biocompatible membranes and materials, and use of ultrapure dialysis fluids and infusion solutions [25,26]; (c) lowers frequency and severity of intradialytic hypotensive episodes resulting in a more rapid decline of RKF and progressive reduction of 24 h urine volume [25,27,28,29]. In selected patients, this gentle progression from conservative therapy to 1WHD with important social, economic, and quality-of-life implications could be a new and interesting choice; furthermore, the 1WHD can last from 6 to 96 months [14], representing a temporary bridge treatment option that can be maintained until more frequent weekly hemodialysis; thus, given the results obtained from our previous studies, it was motivating to extract from the literature studies identified by the term IHD that started and concluded on 1WHD, and evaluated safety, clinical benefits and drawbacks, and impact on the quality of life of enrolled patients.

## 2. Materials and Methods

This systematic review was carried out based on the presence of the term “Incremental Hemodialysis” (IHD). A systematic search of the online databases Medline through PubMed, Embase, Scopus, and the Cochrane Library was performed to include research studies published between 1981 and 31 December 2024. To reduce the risk of bias, all searches were conducted by a sole reviewer. The studies thus identified were based on the application of a series of methodologies for the purpose of initiating 1WHD in selected motivated patients; studies relating to speculative and mathematical models, mixed patient populations undergoing 1WHD + 2WHD, and patients on a 2WHD schedule were excluded. Only studies including the term “Incremental Hemodialysis” in which once-weekly hemodialysis trials had been followed to completion were selected. The PRISMA guidelines were employed to ensure the necessary methodological transparency.

**Figure 1:**
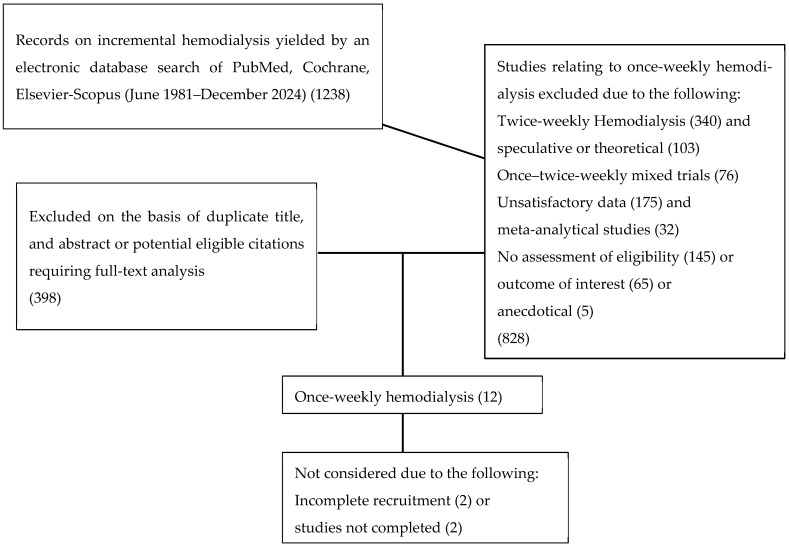
Flowchart of selected studies assessed to identify once-weekly hemodialysis trials.

A thorough analysis of 1238 publications containing the term IHD yielded a total of 12 publications relating to patients on 1WHD (see Figure 1); on further rigorous analysis, these studies were reduced to eight, and of these, only three had been set up with a control group. The baseline details reported in the 8 resulting studies are summarized in Table 1, including the name of the Author, location, and, when provided, the number of recruited patients (male/female), age (years), reference number, baseline creatinine and blood urea nitrogen, mean RKF trend and methods used to calculate the latter, 24 h daily urine volume at baseline and end of the study, causes of renal diseases, nutritional diet with/without supplementation, average duration of 1WHD, hemodialytic adequacy, reasons for drop-out, mortality, cumulative survival, days of hospitalization, and clinical benefits and/or drawbacks. Recruited patients all displayed a good nutritional status with a BMI of 18.5–25 kg/m^2^ and a metabolic steady state. The same criteria were used in three parallel comparison studies. Table 1 shows the PRISMA methodology employed to ensure transparency. Where available, the mean ± SD and/or the median are indicated.

## 3. Results

The studies included were all published between June 1981 and December 2024. They accounted for a total of 299 patients treated with 1WHD with a mean age ranging from 45 to 68 years, in addition to 112 patients included in three comparative studies in which 15 patients had received 2WHD and 87 had received 3WHD. Three studies were conducted in Italy, two in Thailand, one in North America, one in France, and one in Japan. In five studies, average RKF was calculated based on urea + creatinine clearance/2, mL/min/1.73 m^2^ yielding average values of 5.6 ± 2.5, in one using urea clearance mL/min/1.73 m^2^ equal to 5.1 ± 4.0, in two creatinine clearance mL/min/1.73 m^2^ yielding average values of 2.9 ± 1.3, in another CKD-EPI mL/min/1.73 m^2^ yielding values of 6.9 ± 1.4, and in the last, creatinine clearance mL/min/1.73 m^2^ with values of 2.0. Reported dialysis adequacy was measured using single pool Kt/V (spKt/V) or equilibrated Kt/V (eqKt/V), with levels invariably > 1.2. Patients’ BMI ranged from 22.0 to 26.5 kg/m^2^. The average duration of the 1WHD protocol varied between 1.5 and 24 months (15.9 ± 7.6). The dropout rate from 1WHD, defined as progressing to higher frequency weekly hemodialysis sessions across five studies, was 66.9 ± 14.5% (41.9–76.6), reducing to 50.4% at 12 months. In six studies, cumulative survival (1WHD drop-out + deaths) was 35.8 ± 22.3% (17.5–71.4), which decreased to 28.6% at 12 months. In seven studies, the average diuresis volume was 1664 ± 426 at baseline and 802 ± 707 (*p* = 0.018) at the end of the study. A low-protein diet of 0.3–0.6 g protein/kg/day was prescribed in seven studies, of which six prescribed nutritional supplementations with amino acids with or without keto-analogues [8,9,10,12,20,23]; no dietary measures were implemented in two studies [19,20], whilst in another two, patients were allowed to eat freely on the day of their hemodialysis session [13,23,24]. As no statistically significant effects, either positive or negative, were observed across seven studies, the ‘Combined Diet Dialysis Program’ based on a once-weekly hemodialysis protocol should be highlighted [12,14]; indeed, at variance with the findings obtained for a thrice-weekly protocol, the 1WHD schedule yielded the following findings: stable Hb despite a significant reduction in dose of erythropoietin (*p* < 0.001) and reduction in calcium phosphate binders (*p* < 0.002) and use of calcimimetics (*p* < 0.03). In a sample of 75 hemodialysis sessions performed in the context of a 1WHD schedule, inter-dialysis weight gain was 900 ± 923 mL (min. 0, max. 2500 mL), with patients displaying a negative inter-dialysis weight gain in 5 out of 17 hemodialysis sessions (−974 ± −667; min. −200, max −1400), thus dictating a need to reintegrate ideal dry weight during sessions [15]. Following 8–12 days of oligoanuria, urination was reinstated and, in several cases, exceeded 10 L over six extra dialysis days.

The main characteristics of the eight resulting studies are summarized in Table 1, including the name of the Author, location, and, when provided, the number of recruited patients (male/female), age (years), reference number, baseline creatinine and blood urea nitrogen, mean RKF trend and methods used to calculate the latter, 24 h daily urine volume at baseline and end of the study, causes of renal diseases, nutritional diet with/without supplementation, average duration of 1WHD, hemodialytic adequacy, reasons for drop-out, mortality, cumulative survival, days of hospitalization, clinical benefits and/or drawbacks. All recruited patients displayed a good nutritional status, with a BMI of 18.5–25 kg/m^2^ and a metabolic steady state. The same criteria were used in three parallel comparison studies. Table 1 indicates, where detectable according to the statistical methodology used, the mean ± SD and/or the median.

## 4. Discussion

The main goal of a once-weekly hemodialysis schedule is to preserve the safety of patients and quality of life (QoL) for as long as possible. The fundamental basis underlying the inclusion and maintenance of ESKD patients on a 1WHD schedule should be RKF and adherence to a low-protein and caloric-controlled diet.

### 4.1. Hemodialysis Strategies

The depurative efficacy of current extracorporeal dialysis strategies in maintaining adequate control of the uremic metabolism is of secondary importance compared to the contribution provided by a low-protein diet and maintenance of good RKF in the context of IHD, particularly 1WHD. Indeed, more recent high-efficiency dialysis machines, devices, and compatible membranes are capable of achieving adequacy that may even exceed spKt/V and/or eqKt/V > 1.2–1.4, more than sufficient to support a 1WHD schedule with the aim of better-regulating uremia, reinstating euvolemia, and rectifying any electrolyte imbalances. Paradoxically, however, as a downside, the effects of the hemodialysis session on RKF are manifested following the end of the hemodialysis session due to the onset of dialysis-induced hypercatabolism resulting in the generation of toxic small, medium, and large pro-inflammatory and pro-oxidant molecules [30] and the well-known loss of more than 5g of amino acids in the outflow of spent dialysis fluid [31]. Fortunately, this phenomenon wanes over subsequent extra dialysis days. A gradual introduction to a 1WHD schedule will help to avoid an initial and abruptly intense start to HD, particularly in the presence of significant ultrafiltration or intradialytic hypotension, which may lead to a rapid decrease in RKF [32]. Compared to peritoneal dialysis, average daily urine volumes were higher in patients on incremental HD (1WHD–2WHD) than those on conventional HD [22], thus confirming how the transition of ESKD patients from conservative treatment to a 1WHD schedule is better accepted and less traumatic.

### 4.2. Nutritional Considerations

In the context of 1WHD, several studies [13,23,24] allowed patients to eat freely on the day of their hemodialysis session to compensate for the Protein Catabolic Rate corresponding to, or at times exceeding, 1.4 g/kg/day. The need for this nutritional freedom on dialysis day is dictated largely by the energy recovery demand produced by the hemodialysis session itself and by the dialysis-induced loss of amino acids [31]. This extra nutritional “reward” provides patients with an additional reason for embarking on and sticking to the 1WHD schedule. A very low-protein diet (VLP) of 0.6g protein/kg may, however, be required on the six inter-dialysis days, together with amino acid supplementation of mainly essential amino acids (EAAs) and a quota of at least 4g/day of branched-chain amino acids (BCAAs). The decision to supplement amino acids across inter-dialysis days is a rational one, particularly in view of the fact that even with diets providing 0.6 g protein/kg/day, EAAs are decreased by 50%, with EAAs in low-protein diets of 0.4 g protein/kg/day and 0.3 g protein/kg/day being reduced by 33% and 25%, respectively [32,33,34,35]. A series of interesting clinical benefits have been obtained following amino acid supplementation, as demonstrated in a study by Caria S. et al. [12] aimed at preventing, at all costs, the risk of progressive onset of sarcopenia [36] whilst ensuring a calorie intake of at least 25–30 Kcal/day [37]. The maintenance of adequate diuresis preserving phosphaturia even in the presence of GFR < 5 mL/min was underestimated; indeed, considering together levels of phosphaturia + the gastro-intestinal chelating effect of phosphorous chelating agents + low-protein diet of 1.0–1.2 g/kg/day on a 3WHD schedule, a positive phosphate balance of 4.73 g/week is obtained. Accordingly, when adopting a 1WHD schedule with a dietary intake of 0.6 g/kg/day, optimum control of phosphates and hyperphosphatemia characterized by a negative phosphate balance reaching even—3 g/kg/day is undeniably beneficial in reducing the incidence of cardiovascular events associated with the harmful phosphorus metabolism [38].

### 4.3. Motivation, Competence, and Strategies

To effectively maintain a 1WHD schedule, the motivation and awareness of medical and nursing staff are paramount in aiding the transition. The 1WHD program should preferably be implemented in central medical centers to ensure more constant and frequent clinical monitoring. The dialysis team is responsible for effectively monitoring sessions [39] to ensure they proceed as smoothly as possible and, particularly, to limit the onset and intensity of harmful hypotensive episodes that may negatively affect RKF [40,41]. To date, the small number of studies conducted have reported no major episodes of hyperkaliemia and, as yet, no definitive positive or negative correlations in patients receiving amino acid supplementation can be inferred.

An additional benefit may be afforded by the reduced use of the vascular port and the consequent decrease in associated complications [42]. The present review failed to provide irrefutable data relating to mortality rates and days of hospitalization. Only five studies were found to initiate a 1WHD schedule at an RKF of 5.0–10 mL/min/1.73 m^2^, and it is likely unadvisable to consider initiating 1WHD in late-referral patients with an eGFR < 5 mL/min/1.73 m^2^. The best effects are likely obtained both during the transition to 1WHD and in the months immediately thereafter in the presence of an RKF of between 5 and 10 mL/min/1.73 m^2^ and a well-maintained nutritional and metabolic profile. Moreover, to obtain reliable measurements, RKF should be calculated based on the mean of creatinine + urea clearance/2 [43]. Promising findings have been reported in three 1WHD trials, showing a significant decrease in *β*2-microglobulin levels throughout treatment compared to patients on 3WHD [11,19,20,44]. This might have been due to better RKF preservation in incremental HD, potentially resulting in fewer cardiovascular events and lower mortality rates in this group [45].

### 4.4. Limitations

To conclude, patients were assessed with regard to quality of life, anxiety, and depression based on a range of scores that were difficult to combine or compare across studies. No studies reported significant impacts of 1WHD, largely due to the limitations of this review: First, there is a significant paucity of completed studies; second, the timing involved in referring patients to a 1WHD schedule varied across the studies included in the review, thus contributing to significant heterogeneity in results; third, variations in data relating to the definition of outcome and duration of follow-up may confound analysis. Lastly, it should be highlighted that the majority of the studies included in the review were of an observational or retrospective design, which may have introduced limitations in terms of bias and interpretation of causality.

## 5. Conclusions

A once-weekly hemodialysis protocol should be viewed as a bridging strategy between conservative pre-dialysis treatment in patients with ESKD and the onset of a full hemodialysis program geared towards preserving RKF and delaying the need for more frequent hemodialysis sessions. Greater emphasis should be placed on the importance of prescribing a low-protein diet and replacing amino acids lost during hemodialysis with the intent of prolonging RKF and maintaining a good nutritional status and its vastly underestimated ability to facilitate the continuous clearance of small and medium toxic molecules from the body. A clearance index of eq/spKt/V > 1.2–1.4 is safe and easily achievable and should be implemented as soon as a decline in RKF is detected. Further confirmation of the considerations made here should be provided from a wider range of clinical trials investigating the effectiveness of a once-weekly hemodialysis schedule conducted by specially trained and highly motivated nephrologists and nursing staff. In the meantime, however, the term “incremental hemodialysis” should be expanded to include the once-weekly hemodialysis protocol.

## Figures and Tables

**Table 1:** Selected studies on once-weekly hemodialysis.

Study and Location, Methodology		Recruited Patients (M/F)	Age of Recruited Patients, Years	Creatinine at Start, mg/dL	Blood Urea Nitrogen at Start, mg/dL	Residual Kidney Function at Start, mL/min/1.73 m^2^	Average Daily Urine Volume at Start, mL	Average Daily Urine Volume at the End, mL	Method for GFR Measuring	Cause of Renal Disease	Prescribed Hypoproteic Diet, Nutritional Supplement	Duration of Study, months	Dialysis Adequacy Measuring	Average Hospitalization Days per Patient/year During the Study	Causes of Drop Out, % Total Drop-Out, %	Number and Causes of Mortality, %	* Cumulative Survival at the End of the Study	Benefits and/or Drawbacks
Mitch WE et al., 1981 [9] USA Cross-over		7 (5/2)	45	n.r.	75.7 ± 21.5 median:65	3.43 ± 0.98	n.r.	n.r.	average urea creatinine clearances	4GN 2 PN 1 APKD	0.4 g/kg/day + 10 g/day EAAs	3	n.r.	n.r.	14% no dietetic adherence 14% appear of uremic symptoms TOTAL: 28%	n.r.	71.4%	n.r.
Morelli E et al., 1987 [10] Italy Prospective		17 (14/3)	48.5 ± 12	13.5 ± 2.1	n.r.	2.0	1529 ± 420 median: 1450	682 ± 426 median: 500	average creatinine clearances	11 GN, 2 PN, 2 APKD 1 diabetes 1 NASCL	0.3 g/kg/day + EAAs and/or keto- analogues	18.2 ± 17.7 mediana: 11	n.r.	n.r.	35.3% decline of RKF 11.8% patient request 5.9% hydration overload intersession weight gain 5.9% bleeding peptic ulcer 5.9% severe hyponatriemia 5.9% appear of uremic syndrome 5.9% reiterated vomiting TOTAL: 76.6%	5.9% brain hemorrhage	17.5%	average reduction of serum urea + phosphate and serum methylguanidine, lower costs
Locatelli F. et al., 1994, 1998 [11,12] Prospective Italy		69	61 ± 3	12.8 ± 3.5	82 ± 21	2.46 ± 0.9	n.r.	n.r.	average urea creatinine clearances	n.r.	0.4 g/kg/day + EAAs and/or ketoanalogues	24	spKt/V 1.15–1.28	2.84	66.6% no dietary adherence neurological peripheral damage TOTAL > 66.6%	n.r.	24.6%	pogressive worsening of uremic syndrome
Caria S et al., 2014 [13] Bolasco et al., 2016 [15] Italy Prospective	1WHD	38 (25/13)	64.5 ± 13	6.4 ± 1.9	68 ± 18	7.8 ± 1.9 median: 6.2	1983 ± 651	1875 ± 315	average urea creatinine clearances	12 Diabetes, 11 NASCL 7 GN, 3 PN 2 APKD, 2 PN 1 urological	0.6g/kg/day + EAAs + NEAAs free diet on dialysis day	24	eqKt/V > 1.2	3.7 ± 1.5	2.6% kidney transplant 15.8% decline of RKF 13.1% no diet adherence. 2.6% hydration overload intersession weight gain 2.6% recurring hypertension TOTAL: 41.9%	7.9% cardiac events	92.1%	significative manteinance of: good nutritional status and reduction EPO dose, and reduction of phosphate binders dose, and reduction of calciomimetic drugs dose, and decrease of C-Reactive Protein, better quality of life, slowing RKF decline, 50% cost saving
3WHD		30 (19/11)	65.2 ± 11	5.9 ± 1.8	77 ± 15	9.2 ± 4.2 median: 6.8	1472 ± 133	<200 at 6th month	average urea creatinine clearances	13 Diabetes 7 NASC,6 GN 2 PN, 2 APKD	1.2 g/kg/day	24	eqKt/V > 1.2	6.1	none	6.6% cardiac events 3.3% sepsis 3.3% stroke TOTAL: 13.2%	86.8%	manteinance of good nutritional status and good depurative adequacy
Nakao et al., 2018 [21] Japan Prospective		112 (80/32)	median: 63	11.7 ± 4	98.5 ± 30	3.8 ± 0.8	n.r.	n.r.	average creatinine clearances	38 GN, 38 Diabetes 2 PN, 9 APKD 22 NASCL 3 Urological	0.6 g/kg/day salt intake 6 g/day.	24	spKt/V 1.2–1.4	n.r.	33.9% no diet adherence 29.5% decline of RKF 0.9% kidney transplantation TOTAL: 75.9%	11.6% not specified causes	24.1%	slowing the decline of the RKF, 50% saving costs
Takkavatakarn, 2021 [23] Thailand Prospective		11 (7/4)	64.4 ± 15	n.r.	n.r.	5.1 ± 4.0	n.r.	n.r.	average urea clearance	5 GN, 3 NASCL 1 diabetes 1 urological 1 drug caused	0.6–0.8 g/kg/day on non-dialysis days and 1.2 g/kg/day on dialysis day	9.7 ± 6.9	spKt/ > 1.2	n.r.	72.7% decline of RKF 0.9% patient transfer 0.9% respiratory infection 0.9% hydration overload intersession weight gain TOTAL: 75.4%	n.r.	n.r.	better quality of life
Torreggiani M et al., 2022 [20] France	1WHD	30 (17/13)	69.3 ± 14 median: 67.5	6.4 ± 1.9 median: 5.9	median: 102.2	9.1 ± 6.2 median: 8	median: 1850	median: 1825	CKD-EPI	11 NASCL, 8 diabetes 3 GN, 3 APKD 3 NTI, 1 urological 1 genetic	0.6–0.8 g/Kg/day on non-dialysis days and 1.2 g/kg/day on dialysis day	17.2 ± 10.8 median:14	spKt/V > 1.2	3.1	30% hydration overload intersession weight gain 10% decline of RKF 6.6% appear anemia and angor 3.3% appear of hearth failure 3.3% persistent hypercacemia TOTAL: 53.2%	6.6% not specified causes	59.8%	saving costs on 1WHD
	3WHD	67 (38/29)	65.9 ± 13 median: 67	9.1 ± 4.1 median: 8.2	median: 106.4	9.8 ± 3.9 median:5.8	937 ± 641 median: 900	median: 100	CKD-EPI	22 diabetes 13 n.r., 10 GN 12 NASCL 3 NTI 2 SVKD 1 NPP 2 genetic 2 APKD 1 urological 1 Myeloma	three patient 0.6- 0.8 g/kg/day no other specified supplements for prevent malnutrition	24.2 ± 14.9 median: 28.5	n.r.	n.r.	31.3% not specified causes 22.3% improvement of RKF 13.4% decline of RKF 13.4% patient transfer 7.4% kidney transplantation	13.4% not specified causes	0%	n.r.
Praditpornsilpa K. et al., 2024 [24] Thailand open-label, randomized controlled	1WHD	15	58 ± 19.3	n.r.	n.r.	6.9 ± 1.4	2226 ± 279	1119 ± 429	CKD-EPI	n.r.	0.6 g/kg/day + mix of AAs and ketoanalogs on non-dialysis days and 1.0 g/kg/day on dialysis day	12	spKt/V > 1.2	n.r.	n.r.	n.r.	33.3%	n.r.
2WHD		15	61.9 ± 17	n.r.	n.r.	6.2 ± 1.9	1690 ± 986	768 ± 367	CKD-EPI	n.r.	1.0 g/kg/day	12	spKt/V > 1.2	n.r.	n.r.	n.r.	n.r.	n.r.

* Cumulative survival of the CDDP treatment: the composite end point treatment was determined by transition to twice- or thrice-weekly-dialysis plus deaths plus transfert of patients; NASCL Nephroangiosclerosis; EAAs: Essential Amino acids, GN: glomerulonephitis, NASCL: Nephroangiosclerosis, NTI: Tubulo-Interstitial nephropathy, NPP: Cortical Necrosis postpartum, APKD: Adult Policistic Kidney Disease, PN: pyelonephritis, SVKD: Various Severe Vascular Kidney Diseases; AAs: Amino Acids n.r.: no reported by Authors; 1WHD: once-weekly-hemodialysis; 2WHD twice-weekly-hemodialysis; 3WHD thrice weekly-hemodialysis.

## Data Availability

The data presented in this study are available from the corresponding author upon request.
